# The complete Chloroplast genome of *Stachys geobombycis* and comparative analysis with related *Stachys* species

**DOI:** 10.1038/s41598-024-59132-1

**Published:** 2024-04-12

**Authors:** Ru Wang, Zheng Lan, Yongjian Luo, Zhijun Deng

**Affiliations:** 1 Hubei Key Laboratory of Biologic Resources Protection and Utilization (Hubei Minzu University), Enshi, 445000 China; 2 Heilongjiang Bayi Agricultural University, Daqing, 163319 China; 3 Central South University of Forestry and Technology, Key Laboratory of Forestry Biotechnology of Hunan Province, Changsha, 410000 China

**Keywords:** Lamioideae, Phylogenetics, Evolution, Medicinal plants, Molecular markers, Comparative genomics, Evolutionary biology, Systems analysis, Ecosystem ecology, Plant sciences, Plant breeding, Plant cell biology, Plant evolution

## Abstract

Herb genomics, at the forefront of traditional Chinese medicine research, combines genomics with traditional practices, facilitating the scientific validation of ancient remedies. This integration enhances public understanding of traditional Chinese medicine’s efficacy and broadens its scope in modern healthcare. *Stachys* species encompass annual or perennial herbs or small shrubs, exhibiting simple petiolate or sessile leaves. Despite their wide-ranging applications across various fields, molecular data have been lacking, hindering the precise identification and taxonomic elucidation of *Stachys* species. To address this gap, we assembled the complete chloroplast (CP) genome of *Stachys geobombycis* and conducted reannotation and comparative analysis of seven additional species within the *Stachys* genus. The findings demonstrate that the CP genomes of these species exhibit quadripartite structures, with lengths ranging from 14,523 to 150,599 bp. Overall, the genome structure remains relatively conserved, hosting 131 annotated genes, including 87 protein coding genes, 36 tRNA genes, and 8 rRNA genes. Additionally, 78 to 98 SSRs and long repeat sequences were detected , and notably, 6 highly variable regions were identified as potential molecular markers in the CP genome through sequence alignment. Phylogenetic analysis based on Bayesian inference and maximum likelihood methods strongly supported the phylogenetic position of the genus *Stachys* as a member of Stachydeae tribe. Overall, this comprehensive bioinformatics study of *Stachys* CP genomes lays the groundwork for phylogenetic classification, plant identification, genetic engineering, evolutionary studies, and breeding research concerning medicinal plants within the *Stachys* genus.

## Introduction

The genus *Stachys* encompasses a diverse collection of herbaceous and shrubby plants, involving around 300 species distributed across temperate and tropical regions worldwide, except for Australia and New Zealand^1^. *Stachys* species have been found to hold extensive medicinal value and have a long history of use, rendering them highly valuable for medicinal research and development^1^. Presently, research on *Stachys* species primarily focuses on examining their chemical composition and pharmacological effects^2^. For instance, the chemical composition, extraction of active compounds, and pharmacological effects of *Stachys* plants have been frequently analyzed, with flavonoids, diterpenes, fatty acids, and phenolic acids identified as primary secondary metabolites^3–5^.

Correctly identifying species is fundamental to biological research. However, *Stachys* species present challenges due to frequent geological changes, climate variations, and interspecific hybridization. They exhibit extensive variation in morphological and cytological characteristics^6^. However, some species are also highly polymorphic and vaguely delimited, making them challenging taxonomic units in plant classification and phylogenetics^7^. Research on the phylogeny of *Stachys* mainly focuses on the classification of Stachydeae. Although *Stachys* species exhibit extensive variations in morphology and cytological characteristics^8^, they typically have tubular to campanulate or nasal shaped calyx with equally or nearly equally short teeth at the calyx apex, and there may be a ring or hair ring inside the corolla tube^9^. Pollen characteristics play an important role in species identification in cytology. However, a study by Salmaki et al.^8^ on the pollen of 30 taxa of the *Stachys* genus and a closely related genus *Sideritis montana* distributed in Iran found that while some *Stachys* plants exhibit distinctive pollen morphological features, they cannot be completely differentiated based on pollen morphology alone. Hence, relying solely on morphological and cytological analysis is insufficient for such a complex genus^10^. The development of sequencing technologies and the expansion of molecular databases have rendered them powerful tools for exploring the differentiation and interspecific relationships of *Stachys* species. In previous studies, molecular evidence such as ISSR^10,11^, RAPD^10^, and DNA fragments including the nrITS region^7,12,13^ and cpDNA fragments^9,14^ has been utilized to reconstruct the phylogeny of Stachydeae. For instance, Salmaki et al.^7^ conducted nuclear (ribosomal ITS) and plastid (*trn*L intron, *trn*L-*trn*F spacer, *rps*16 intron) DNA sequence analysis of 143 species in the Stachydeae tribe, and found that both nuclear and plastid DNA data supported the monophyly of the Stachydeae tribe. Phylogenetic studies of *Stachys* plants based on ribosomal and plastid DNA data^15,16^ demonstrated it as an incomplete clade divided into two distinct lineages. The center of diversity for the first lineage is located in the eastern Mediterranean region and has migrated over time to West Asia, Western Europe, Macaronesia, and sub-Saharan Africa. Meanwhile, the second lineage includes Hawaiian mints, Suzukia, all New World *Stachys *species, and some Old World species^17^. Berumen et al. used cpDNA regions for plant phylogenetic reconstruction and suggested reducing the number of members in the* Stachys coccinea* complex to three species, i.e., *S. coccinea*, *Stachys lindenii*, and *Stachys albotomentosa*. Meanwhile, their original ranges, including *S. pacifica*, *Stachys manantlanensis*, *Stachys torresii*, and* Stachys jaimehintonii *should be retained as varieties of *S. coccinea*^9^. In addition, using nrITS DNA region sequences, Özal et al. found the phylogenetic relationship between newly discovered *Stachys *species and their close relatives, and the newly discovered *Stachys istanbulensis *and its relatives *Stachys recta* and *Stachys atherocalyx* formed a branch^17^.

Chloroplasts are semiautonomous organelles unique to higher plants and some algae, which are also present in a few protists^18^. The CP genome, known for its sequences short and relative independence from the nuclear genome, holds a crucial position as the second largest genome in the plant kingdom^19^. Within angiosperms, chloroplasts harbor a wealth of genetic information and present distinctive characteristics such as small relative molecular weight, simple structure, moderate evolutionary rate, low mutation rate, genetic stability, low cost, and ease of development of microsatellite sequences^20^. partially compensating for the limitations of mitochondrial and nuclear genomes^21^. Moreover, CP genome research considerably triggers studies on single nucleotide polymorphisms (SNPs), phylogenetics^22^, and DNA barcoding^23^, while facilitating investigations into the geographical origins of domesticated crops^24–26^. In recent years, CP genomes have found extensive application in classification studies at the genus and even family levels for various plants^27,28^. Chloroplast genomes have been widely applied in taxonomic studies at the genus and even family levels of various plants. For instance, based on the sequences of 79 chloroplast protein-coding genes, Zhao *et al*.^29^ selected 175 species from 79 genera in the Lamiaceae family, proposing a new classification system with 12 subfamilies and 22 tribes within Lamiaceae. Li* et al*.^30^ conducted the first examination of the structural patterns of Pholidota plastomes, providing novel insights into the phylogenetic relationships within Pholidota and its related genera through comprehensive genome data analysis. These studies have demonstrated that utilizing chloroplast genomes can effectively enhance phylogenetic resolution. However, only brief reports on the chloroplast genomes of *Stachys* species, specifically *Stachys sieboldii*^31^and *Stachys japonica*^32^ have been published, leaving room for in depth analysis of the chloroplast genomes.

*Stachys geobombycis* is a perennial herbaceous plant of the Lamiaceae family in the *Stachys* genus, mainly found in various provinces in southern China. The main edible part of *S. geobombycis* is its underground tuber, which has beneficial effects such as clearing heat and detoxifying, promoting blood circulation and removing stasis, dispelling wind and dampness, nourishing Qi and blood, as well as promoting health and beauty. It is often used as a medicinal resource and cooking ingredient.^33^. Currently, studies on *S. geobombycis *have been rarely reported, with the focus placed on chemical composition and pharmacological properties^34–36^. It should be noted that some morphological characteristics of the *Stachys* genus exhibit minimal differentiation, and phenotypic traits show instability^1^. For example, based on phenotypic traits alone, it is difficult to distinguish* S. geobombycis *from* Stachys sieboldii* and *Stachys affinis*, which also rely on underground tubers for food. This poses significant challenges to the agricultural production of *S. geobombycis*. Besides, this characteristic hinders rapid and precise classification based solely on morphological attributes, posing challenges to species identification within the *Stachys* genus^37^. The rapid development of molecular biology and genomics provides valuable genetic information for systematic evolution and species identification in the study of plant chloroplast genomes^38^. However, the chloroplast genomes of the *Stachys* genus have been relatively underrepresented, and there is a lack of comprehensive and collaborative research on chloroplast genome datasets^31,32,39^. To this end, the complete sequence of the chloroplast genome of *S. geobombycis *was hereby collected, and compared with the chloroplast genomes of 7 closely related species through comparative genomics. The present study was carried out for the following purposes: (1) to compare the characteristics of the chloroplast genome of *Stachys* genus and detect differences among 7 species; (2) to identify repeat sequences, simple sequence repeats and genetically variable regions, and select divergence hotspots as candidate DNA markers; (3) to explore their IR expansion and contraction, and estimate genes selective pressure and codon usage; (4) to reconstruct phylogenetic relationships of *Stachys *species based on the cp genome alignments, and verify their phylogenetic position within *Lamioideae*. Overall, this study is expected to provide theoretical basis for the genetic breeding and phylogenetic research of *Stachys* plants.

## Results

### Characterization of the CP genome structure of *Stachys* species

**Figure 1:**
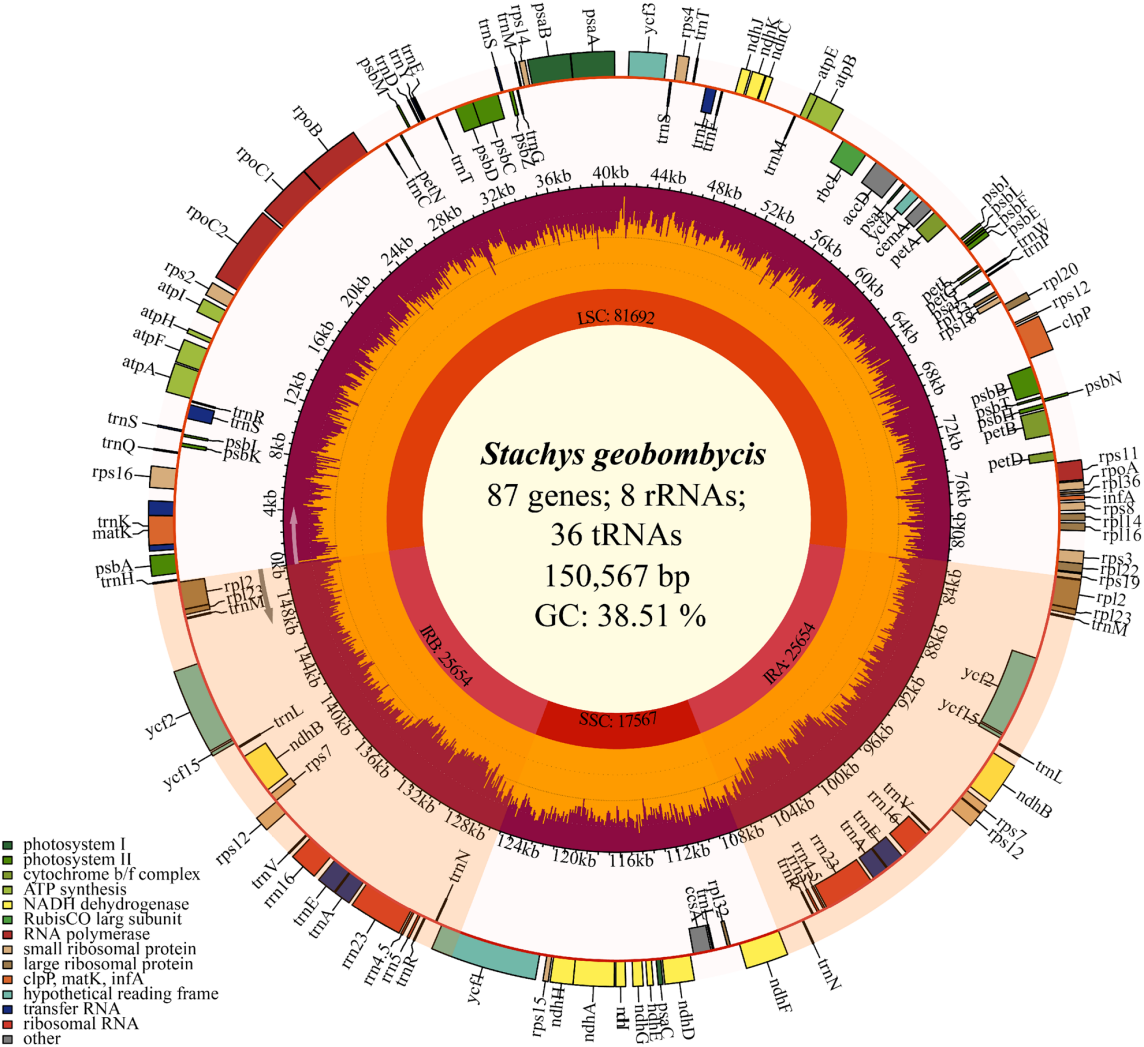
The circular map of *S. geobombycis* CP genome. The second circle displays the gradient GC content distribution of the genome, where the outermost circle represents the zero level. Gene names and their codon usage bias are in the outer layer of the map. The GC content specific to each gene is visually represented by shaded areas relative to their proportions. The inner genes are shown with arrows indicating their clockwise transcription directions, while the outer genes are indicated with arrows in an anticlockwise direction.

The CP genome of *S. geobombycis* was submitted to the GenBank database with the accession number OR327475 maintained by the National Center for Biotechnology Information (NCBI). The total length of the chloroplast (CP) genome for *S. geobombycis* is 150,567 base pairs (bp), and it has been sequenced with an average coverage depth of 1612.19x (Supplementary Fig. 1). It possessed a unique quadripartite structure comprising an LSC (large single copy), an SSC (small single copy), and a pair of IRs (inverted repeats) measuring 81,692 bp, 17,567 bp, and 25,654 bp, respectively (Fig. 1). In the CP genome of* S. geobombycis*, there were a total of 131 predicted functional genes. Among these, there were 110 unique genes, which could be further classified into different groups, including 8 rRNA genes, 36 tRNA genes, and 87 protein coding genes. Besides, the protein coding genes were divided into 4 groups based on their functions. The first group consisted of 45 photosynthetic genes, the second group included 63 self replication expression related genes, the third group contained 6 other genes, and the fourth group comprised 7 genes of unknown function (Supplementary Table 1). Additionally, 13 in the genome contained intron sequences. Among these, the genes *clp*P and *ycf*3 contained two introns each, while the remaining genes had only one intron each (*rps*16, *atp*F, *rpo*C1, *pet*B, *pet*D, *rpl*2, *ndh*B,* ndh*A, *ycf*1, *ndh*B, and *rpl*2).

The CP genomes of the 8 studied *Stachys* species exhibited a characteristic circular double chain structure, varying from 149,523 to 150,599 bp in size (Table 1). The *Stachys* plastomes displayed the conventional quadripartite architecture, consisting of a LSC region (81,156–81,743 bp) and a SSC region (17,057–17,977 bp) separated by two IR regions (25,250–25,666 bp). The total GC content of all 8 CP genomes was similar, ranging from 38.36 to 38.53%. Variation in the number of genes among individuals was observed in some species. For instance, *S. japonica* and *S. coccinea* were reported to possess 123 and 133 genes, respectively^32^. To mitigate the influence of refer-ence genomes and annotation software, the plastid genomes of 7 *Stachys* species obtained from NCBI were re-annotated utilizing the PGA (Plastid Genome Annotator) program^40^ and Geneious v11.0.336^41^, with *S. geobombycis* taken as the reference. The analysis revealed that all plant genomes were annotated with a total of 131 genes (Except* S. coccinea*), comprising 87 protein coding genes, 8 ribosomal RNA genes, and 36 transfer RNA genes. The gene count and types were consistent with those of *S. geobombycis*, indicating strong conservation of the *Stachys* CP genome in genetic evolution.

**Table 1:** Comparison of CP genome features of eight *Stachys* species.

Feature	*S. geobombycis*	*S. affinis*	*S. byzantina*	*S. chamissonis*	*S. coccinea*	*S. japonica*	*S.* *sylvatica*	*S. palustris*
GenBank accession no.	OR327475	MT241264	KU724141	KU724138	NC_029823	MT554703	MT580001	KU724140
Genome size	150567	149523	149749	150254	150275	150599	150167	150559
Large single copy (LSC)	81692	81156	81272	81743	81741	81701	81663	81642
Small single copy (SSC)	17567	17057	17977	17552	17563	17566	17560	17560
Inverted repeat (IR)	25654	25655	25250	25495	25501	25666	25530	25654
Number of protein-coding genes	87	87	87	87	88	87	87	87
Number of tRNAs	36	36	36	36	37	36	36	36
Number of rRNAs	8	8	8	8	8	8	8	8
G+C (%)								
Large single copy (LSC)	36.76	36.85	36.86	36.75	36.72	36.77	36.79	36.79
Small single copy (SSC)	32.46	32.44	32.51	32.59	32.47	32.44	32.53	32.53
Inverted repeat (IR)	43.38	43.38	43.34	43.43	43.24	43.37	43.38	43.38
Total genome	38.51	38.43	38.48	38.42	38.43	38.53	38.36	38.5

### Codon usage analysis

**Figure 2:**
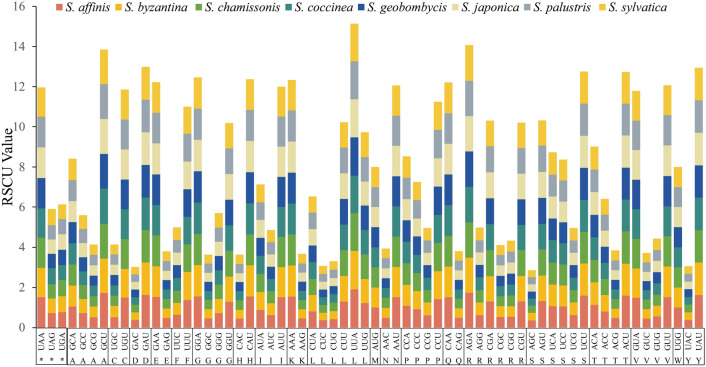
Codon content for the 20 amino acids and stop codons of CDS of the *Stachys *species CP genome.

In order to investigate codon usage patterns and nucleotide composition in the 8 *Stachys* plastomes, amino acid frequency, codon usage number, and the relative synonymous codon usage (RSCU) were analyzed and summarized^42^. The results showed that all 58 homologous protein-coding genes in these species consisted of 64 codons, and encoded 20 amino acids, including three stop codons (UAG (*), UAA (*), UGA (*)) (Supplementary Table 2). The number of encoded codons varied between 21,330 and 22,935 across the species. While the overall count of codons exhibited minimal variation, the types of codons and amino acids remained consistent. Besides, the RSCU value was used to measure the association between the observed frequency and the anticipated frequency of a particular codon. Out of the 64 codons, excluding the three stop codons and the unbiased methionine (Met) and threonine (Thr) (RSCU = 1), 31 codons displayed a preference with RSCU values exceeding 1, indicating a higher priority for these codons. Among them, the AUU codon for Leucine (Leu) had the highest frequency as indicated by an average RSCU value of 1.89. The remaining 31 analyzed codons showed relatively low bias, with RSCU values less than 1 (Fig. 2). The codons in the eight CP genomes of *Stachys *species exhibited a preference for A/T bases and A/T-ending codons, as evidenced by the GC and GC3s content being below 0.5. Besides, analysis of codon adaptation index values and an effective number of codon values revealed a minor tendency toward biased codon usage in the *Stachys* species. The frequency of optimal codons was relatively low. Furthermore, the hydrophobicity and aromaticity of the protein, as measured by Gravy and Aromo respectively, had a minimal effect on the observed bias in codon usage.

### Identification of repeat elements

**Figure 3:**
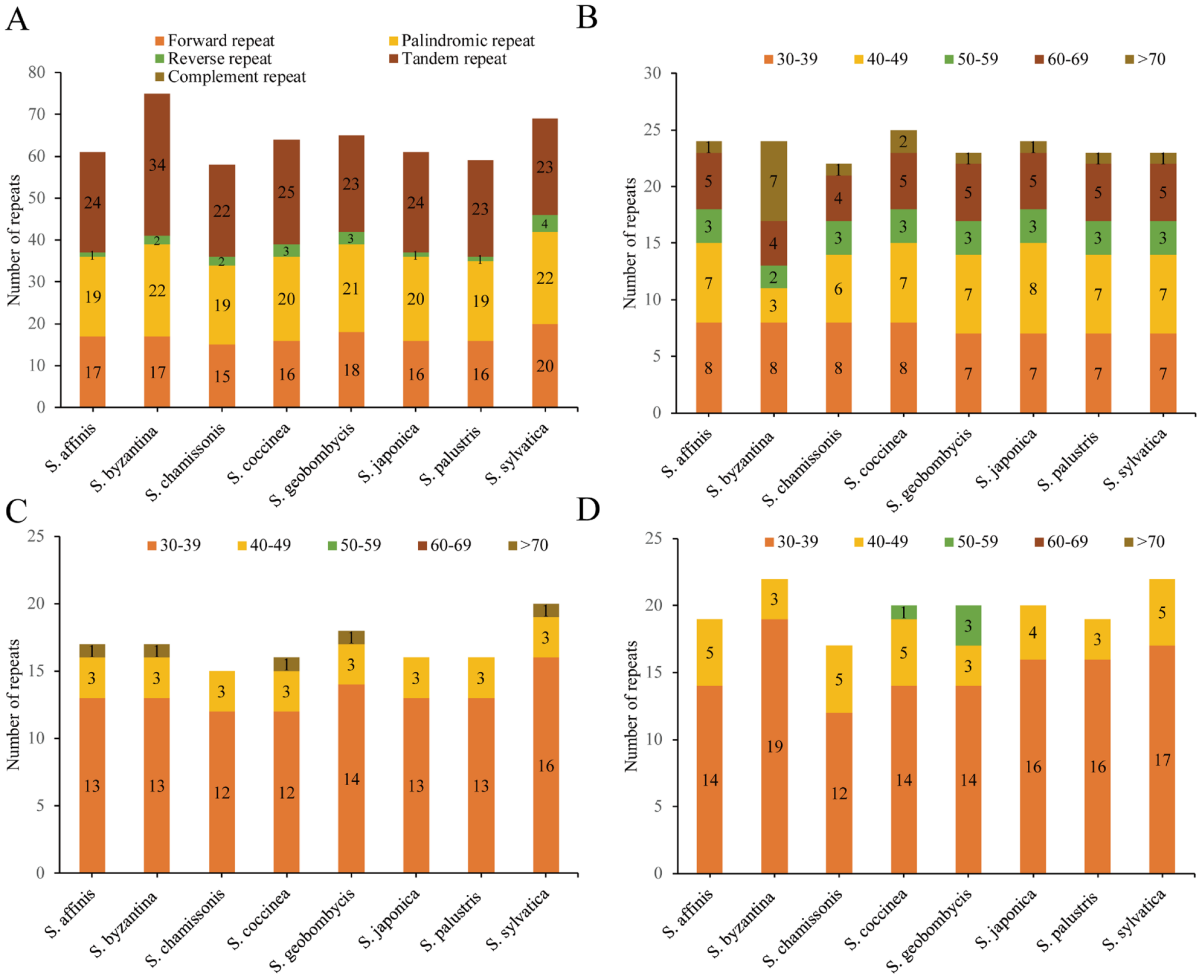
Repeat sequences analysis in 8 *Stachys* plastomes. (**A**) The types of 8 CP genomes; (**B**) displays the number of tandem repeats in 8 CP genomes; (**C**) represents the number of palindromic repeats in 8 CP genomes; (**D**) illustrates the number of forward repeats in 8 CP genomes. The repeats of varying lengths are depicted in different colors, with the y-axis representing the number of repeats.

Among the 8 analyzed CP genomes, a total of 512 long repeats were detected, comprising 135 forward repeats, 198 tandem repeats, 17 reverse repeats, and 162 palindromic repeats. The analysis revealed a varying number of repeated sequences in the 11*Stachys* CP genomes, ranging from 58 in *Stachys chamissonis* to 75 in* Stachys byzantine*. Tandem repeats were the most common type among these repeats, accounting for 33.3–45.43% of the repeats and varying from 22 (*Stachys affinis*, *S. chamissonis*, and *Stachys palustris*) to 34 (*S. byzantine*, *S. palustris*), followed by palindromic repeats (31.34–32.78%), ranging from 19 (*S. chamissonis*) to 22 (*Stachys sylvatica*), and then by for-ward repeats (22.67–28.98%)ranging from 15 (*S. chamissonis*) to 20 (*S. sylvatica*) (Fig. 3A). Meanwhile, the length of long repeats differed across the 8 sequenced CP genomes, with the majority falling within the 30–49 bp range (Fig. 3B–D).

**Figure 4:**
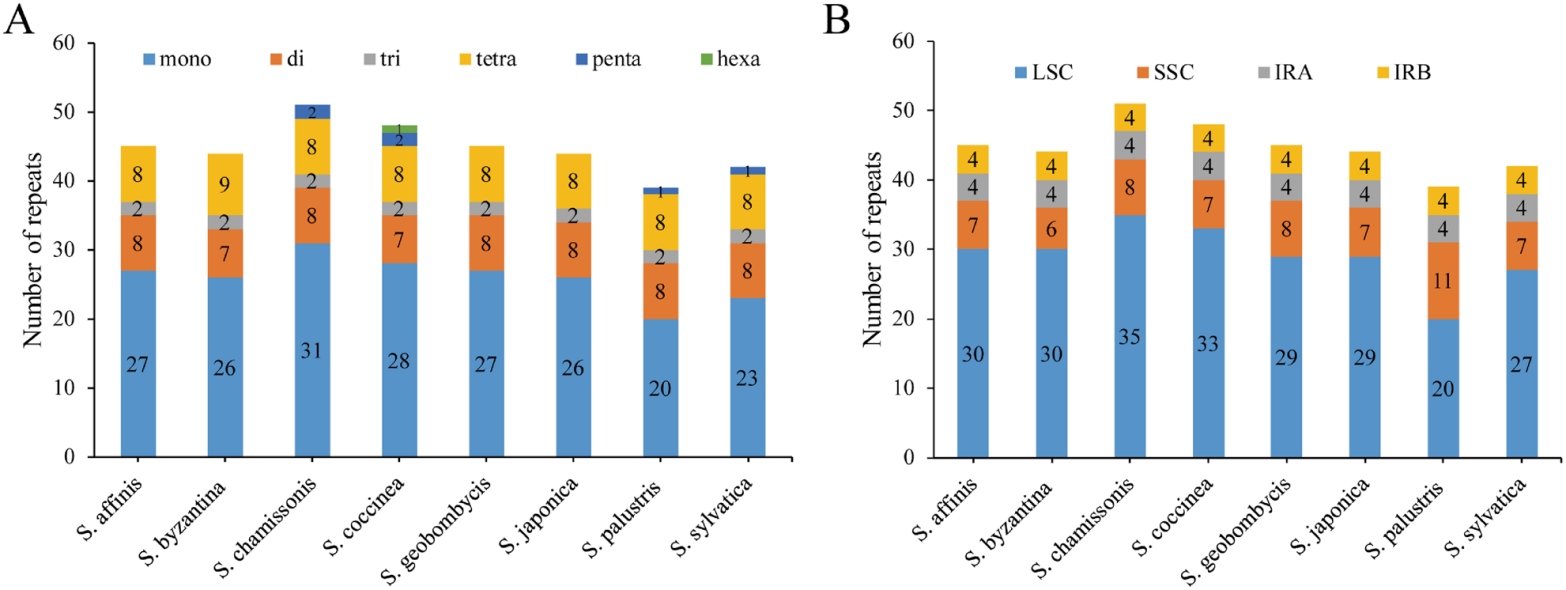
Analysis of the number and type of SSRs in 8 *Stachys* species plastomes. SSRs with different types are visually represented using different colors. A depicts the analysis of the number and type of SSRs in 8 *Stachys* species plastomes; B shows the distribution of different types of SSRs in the chloroplast genome.

The highest number of SSRs was identified in *S. chamissonis* (51), followed by *S. coccinea* (48) (Fig. 4A), while the smallest number of SSRs, 152, was identified in *S. palustris*. The most frequently observed SSR type was mononucleotide repeats, ranging from 20 to 31. All species exhibited Mono-, di-, tri-, and tetra-nucleotide repeats, while penta-nucleotide repeats were only observed in *S. coccinea*, and hexa-nucleotide repeats were present in *S. chamissonis*, *S. palustris*, and *S. sylvatica *species. Furthermore, as shown in Fig. 4B, the SSR distribution was mostly located in the LSC region (51.28–68.75%), followed by the SSC (13.36–28.21%) and IR regions (15.68–20.51%).

### IR contraction and expansion in the *Stachys* CP genomes

**Figure 5:**
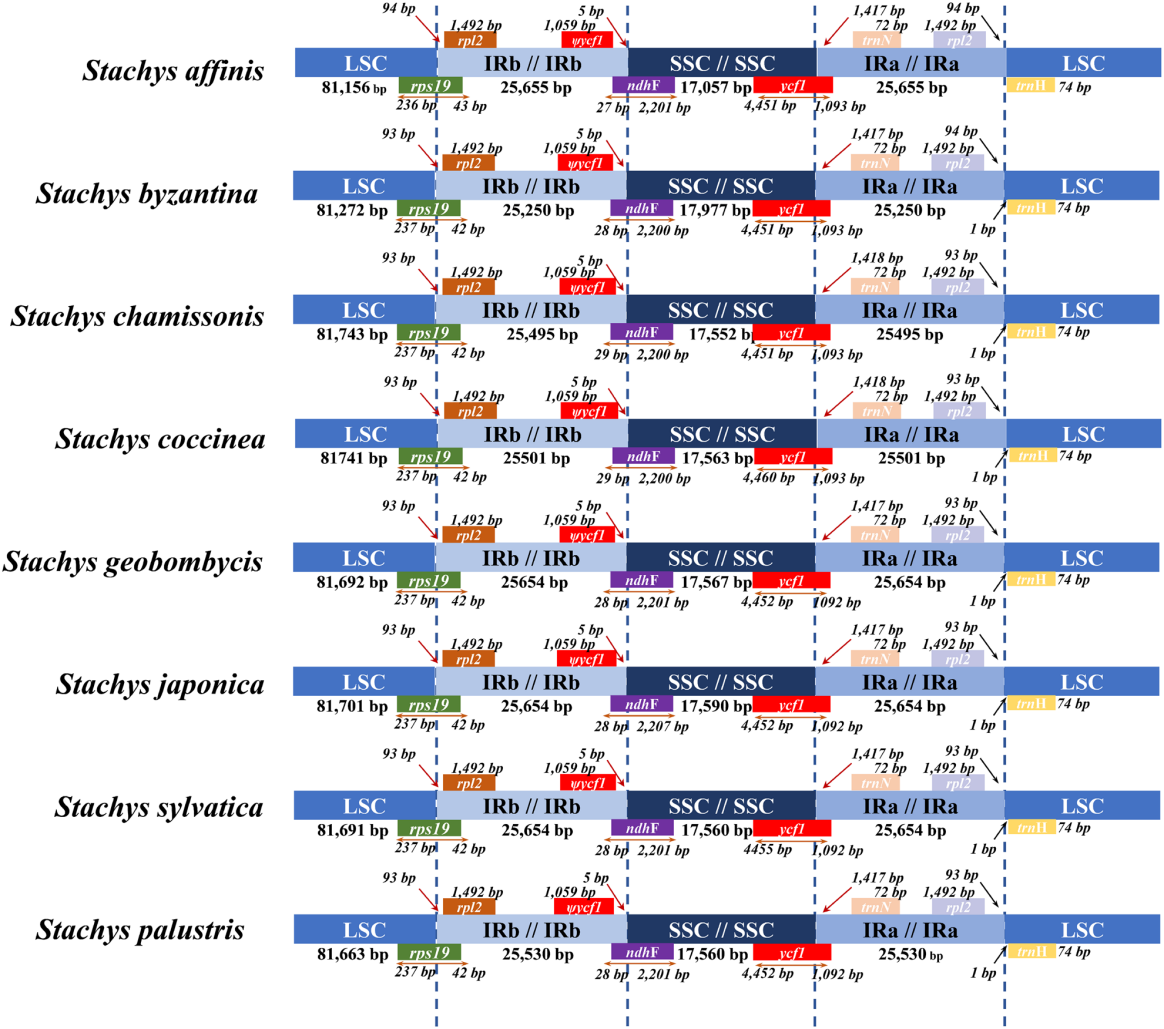
Comparison of chloroplast genome structure in 8 *Stachys species*.

The genotypes of the IR-LSC and IR-SSC boundaries were almost identical, and the lengths of the IRs in the 8 *Stachys* cp genomes were relatively conserved (25,250–25,655 bp) (Fig. 5), involving no obvious amplification or contraction events. At the LSC-IRB boundary, a fragment of *rps*19 gene was detected, with no difference in the 8 CP genomes. At the IRB/SSC boundary, the pseudogene *ycf*1 was situated 5 bp from the left side of LSC-IRB, with the *ndh*F gene extending across the LSC region for 28–29 bp, overlapping with the *ycf*1 pseudogene. At the SSC/IRA boundary, the *ycf*1 gene was consistently found in all 8 cp genomes and spanned 1092–1093 bp across the IRA boundary. The *trnH* gene was located 1417–1418 bp from the SSC/IRA boundary within the IRA region. The *rpl*2 and *trn*H genes were positioned between the IRa-LSC boundary, with *rpl2* located on the left side of the boundary for approximately 93–94 bp, and *trn*H on the right side, spanning 0–1 bp.

### Comparative genomic analysis

**Figure 6:**
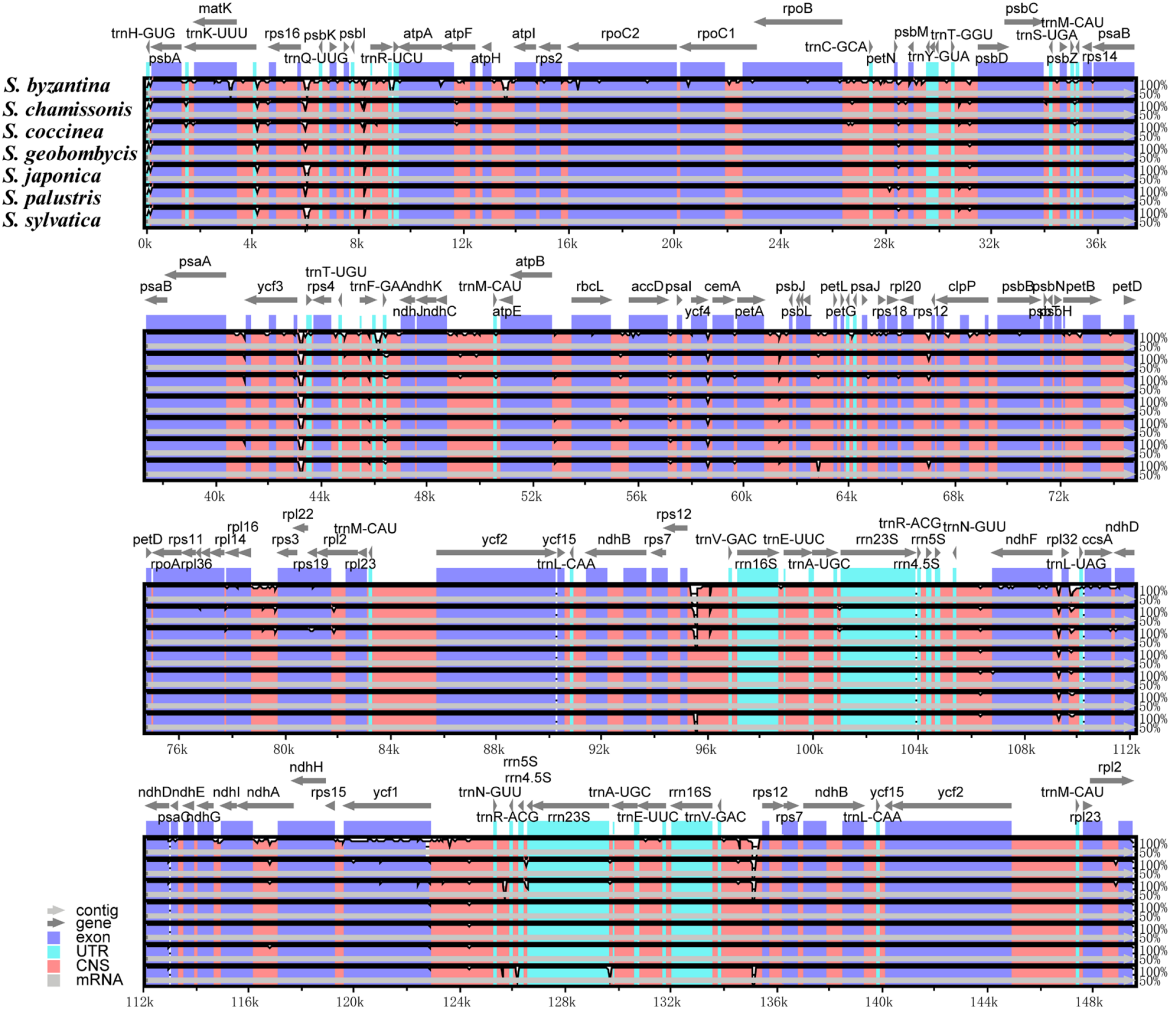
Visualization of alignment of 8 chloroplast genomes using *S. affinis* as a reference. The vertical scale depicts a range of 50–100% and represents the percentage of similarity.

The multiple genome alignment method of chloroplast genomes detected only one locally collinear block (LCB) among the 8 sequences (Supplementary Fig. 2). The types, quantities, and arrangement of all genes remained highly consistent within the genus. The chloroplast genomes of this genus were completely collinear, without any occurrence of rearrangement or recombination events, further indicating a high level of conservation. In order to elucidate the sequence differences among *Stachys* plants, the analysis was conducted with the *S. affinis* CP genome as reference in the mVista software (Fig. 6), and the results indicated no significant alterations such as large fragment inversions, duplications, or other structural changes of the 8 plastomes. The genomes exhibited a high level of collinearity, indicating a consistent evolutionary conservation at the genomic level. Besides, sequence differences were higher in the LSC and SSC regions compared to the IR region. Consistent with previous studies on angiosperm CP genomes, the coding regions exhibited a higher degree of conservation compared to the non-coding regions.

**Figure 7:**
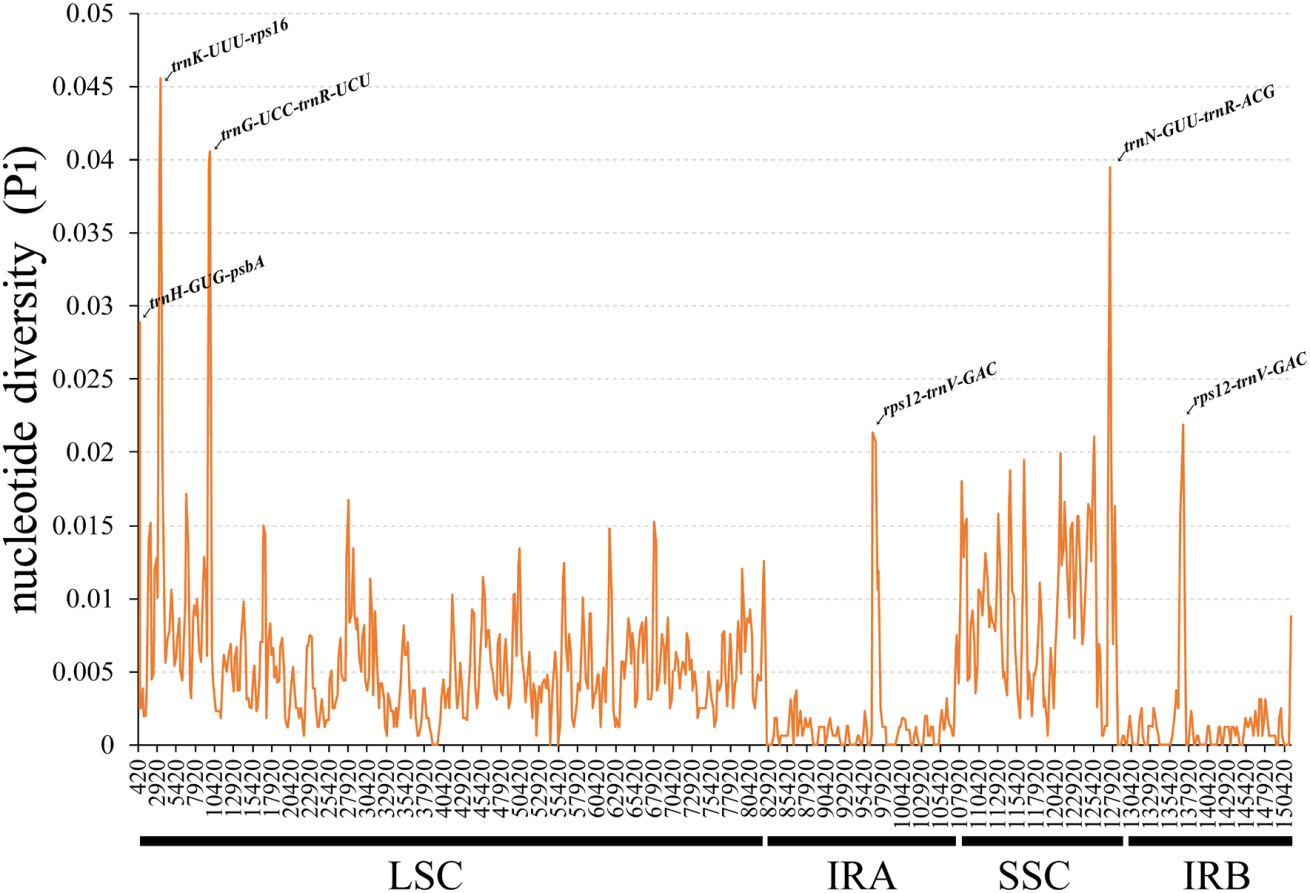
A comparative analysis of nucleotide variability, measured by Pi values, across the 8 CP genomes using a sliding window approach (window length: 800 bp; step size: 200 bp). The X-axis indicates the midpoint position of each window, while the Y-axis represents the nucleotide diversity within each window.

Subsequently, multiple sequence alignments of the complete CP genomes were conducted, and the nucleotide diversity within a 600bp window was then calculated for all 8 CP genomes, The observed values ranged from 0 to 0.07746 (Fig. 7), and the analysis showed the highest difference level of the SSC region compared to other regions (Average pi = 0.007222681). The LSC region had a range of PI values from 0 to 0.04554, with an average of 0.005641219, while the IR region had the lowest Pi values, ranging from 0 to 0.02134, with an average of 0.001354375, indicating the IR region as the most conserved region, which was consistent with the results shown in Fig. 6. In addition, 5 hotspot regions (Pi > 0.02, average = 0.037), including *trn*H-GUG*-psb*A, *trn*K-UUU*-rps*16, *trn*G-UCC*-trn*R-UCU, *trn*N-GUU*-trn*R-ACG, and *rps*12*-trn*V-GAC, were identified. These sequences could serve as potential markers for further phylogenetic reconstruction and species identification in *Stachys* species.

### Phylogenetic analysis

**Figure 8:**
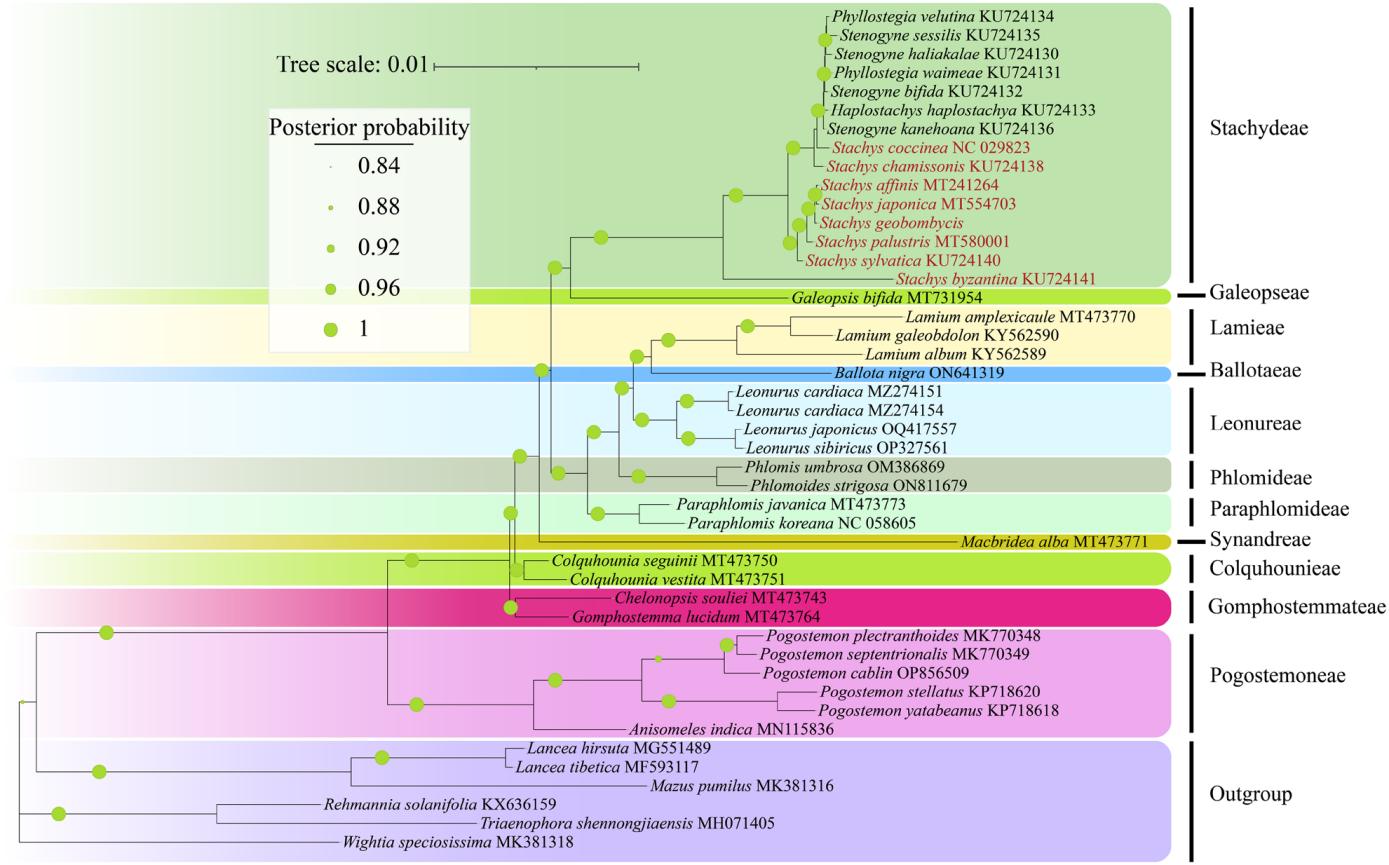
Phylogenetic relationships among 46 plant species based on CP genome. The Phylogeny is inferred by employing the BI method and concatenating the sequences of protein-coding genes from all the species. The support for each branch is indicated by green circles, while posterior probability values represent the level of support.

In order to ascertain the evolutionary position of *Stachys* within the Lamioideae subfamily, the complete CP genome sequences of 32 other sequenced CP genomes were aligned by multiple sequence alignments. Phylogenetic reconstruction was carried out using the ML (maximum likelihood) method and BI (Bayesian inference) analysis based on 40 complete CP genomes of Lamiaceae (Fig. 8 and Supplementary Fig. 3). Both methods produced nearly identical tree topologies with high support values of Bayesian posterior probability (PP) and maximum likelihood bootstrap support (BS) in each branch. Almost all nodes on the phylogenetic tree received strong support (PP/BS = 1/100), even though some clades represented a limited number of species. In the phylogenetic tree, Pogostemoneae was identified as the earliest diverging branch, followed by Gomphostemmatae, Colquhounieae, Synandreae, Betoniceae, Galeopseae, Stachydae, Aparlomideae, Phlomideae, Leonureae, Marrubieae, Leucadeae, and Lamieae. All *Stachys* samples were nested within Stachydeae, with* S. geobombycis* being closely related to *S. japonica *and *S. affinis*. *S. byzantina* from Western Asia was located in the basal clade of Stachydeae, while species *S. chamissonis *and *S. coccinea* from North America were in the basal clade of other Stachydeae species. Besides, the remaining *Stachys* species from East Asia were grouped together, forming a well supported branch, which was consistent with previous reports^7,29^.

## Discussion

### Structure characteristics of the CP genome in the *Stachys *species

The chloroplast (CP) genome of plants is a valuable resource for studying intra and inter species evolution and developing molecular markers^18^. In this particular investigation, CP genome sequencing and annotation of* S. geobombycis *officinale were conducted, and its features were compared with those of 7 other *Stachys* species. The findings demonstrate that the *Stachys* CP genome, like other angiosperms, consisted of a circular double stranded DNA molecule with a conserved quadripartite structure^38,43^. This structure included a large single-copy region (LSC), a small single copy region (SSC), and two inverted repeat regions (IRs). Notably, the *Stachys* CP genome exhibited significant conservation and similarity to previously reported plastomes within the Lamiaceae family^44,45^.In terms of size, the *Stachys* CP genome ranged from 14,523 to 150,599 bp, displaying a difference of 1077 bp across different genomes, which indicated the relatively stable and conserved nature of the *Stachys* CP genome. The major variation in genome size could be attributed to the varying length of the LSC (558 bp), suggesting that changes in LSC length primarily drive the variation in genome length. Overall, the GC content of the 8 *Stachys* samples ranged from 38.36 to 38.53%. Generally, higher GC content contributed to the stability and complexity of genome sequences^46^. Interestingly, the present study revealed a lower GC content in the LSC and SSC regions of the CP genome than in the IR region, which was a characteristic feature observed in angiosperms. This discrepancy was primarily attributed to the presence of four high GC content rRNA genes in the IR region^47^. Furthermore, the gene composition, protein coding genes, tRNA, and rRNA in the *Stachys* CP genome exhibited high similarity. This conservation of plastomes was consistent with previous reports in various angiosperms, such as Malvaceae and Araceae, where identical gene content and order were observed^48–50^. Similarly, a high degree of conservation in the CP genomes of *Stachys* species was hereby. Various molecular mechanisms, including maternal inheritance, rarity of plastid fusion, and active repair mechanisms, were found to contribute to the maintenance of CP genome conservation in *Stachys*^51^, resulting in the typically conservative nature of *Stachys* plastomes.

### Analysis of repetitive sequences and codon bias

In terms of evolutionary rate and pattern, the synonymous codon usage bias (SCUB) in plant CP genomes differs from that of mitochondrial and nuclear genomes. In addition to being influenced by DNA sequence mutation pressure and natural selection affecting gene translation^52^, SCUB in plant chloroplast and mitochondrial genomes is also associated with other factors such as tRNA abundance, strand specific mutation bias, gene expression levels, and gene length^53–57^. These influencing factors have been widely used to explain variation in codon usage among species and within genomes^58^. In this study, specific codons were found to be more frequently used than other synonymous codons in the nucleotide sequences of protein coding genes in the *Stachys *species CP genomes, consistent with previous reports. Besides, no distinct species specific features were observed in codon usage levels among the 8 *Stachys *species. Relative Synonymous Codon Usage (RSCU) is often used to reflect codon bias. The GC content of the CP genomes is a result of the balance between mutation pressure and adaptation, which is one of the most common influences in the formation of codon usage bias^59^. Although synonymous mutations of the third base of a codon do not change the amino acid type, it is still considered an important feature in determining the amino acid type. Therefore, GC3 is often used as a significant indicator of codon preference^60^. Herein, the *Stachys *species CP genomes, all optimal synonymous codons (RSCU>1), except UUG and UCC, had A or U at the end, leading to a preference for A/T bases throughout the genome. Chloroplast transformation has made significant strides in various domains, including crop salt tolerance, drought resistance, and herbicide resistance in recent years. In conducting chloroplast gene breeding research, it is crucial to consider the stability of chloroplast genes and their genetic diversity^54^. RSCU can affect gene expression by regulating the accuracy and efficiency of translation, with stronger RSCU values associated with higher gene^61–63^. In chloroplast gene expression vector design, optimizing codons according to their bias can boost the expression levels of inserted genes in the CP genome. Additionally, known codon usage patterns can help predict the expression and function of unknown genes^64^. The identified codons with RSCU>1 can serve as efficient indicators for detecting the expression levels of hypothetical genes or open reading frames. They are also valuable for designing primers and introducing point mutations in agricultural breeding research.

SSRs can be found in various regions of both prokaryotic and eukaryotic organisms, including both coding and noncoding regions^65^. Microsatellite sequences are favored markers in plant genetics and breeding research due to their variability, simplicity in utilization, detectability, and repeatability. They have been extensively employed in studies related to biodiversity and population genetic^66^. In this study, a total of 358 SSRs were detected in the CP genomes of 8 *Stachys* species, and the majority of SSRs were found in the LSC region, which might be correlated with the length of the LSC region. Additionally, they were predominantly composed of A/T bases, consistent with the AT richness observed in the entire CP genome^19^. Moreover, repeat sequences are essential for studying insertions, deletions, and replacements, and they are highly abundant in the chloroplasts of *Stachys* members^67^. In this study, a total of 512 long repeat sequences, encompassing 4 types, were identified. Overall, the SSRs and long repeat sequences identified in this study provided useful information for further research on molecular marker development, population genetics, evolution, breeding, species identification, and conservation studies in the *Stachys *species^68^.

### Comparative genomics and highly variable regions analysis

In angiosperms, it is common for the Inverted Repeat (IR) regions in the CP genome to undergo expansion and contraction. This phenomenon often results in size variations, gene duplications or deletions, and the generation of pseudogenes^69^. Abnormal expansions of the IR region, transferring a large number of genes from the Small Single Copy (SSC) region to the IR region, have been observed in some taxa, such as* Paphiopedilum *^70^, *Bidens*^71^, and *Pilea*^56^. In this study, significant similarities were found in the expansion and contraction of the IR regions in* Stachys*, with highly consistent distribution and positioning of genotypes in these regions. The IR boundaries were relatively stable, consistent with previous reports in Lamiaceae^49^. The movement of the IR/SSC boundary in* Stachys* always leads to an increase in the length of the IR region. Overall, the conservation of the IR region in *Stachys* may contribute to its overall length and structural stability.

DHighly variable regions with informative sites served as DNA barcodes, enabling the construction of phylogenetic trees and identification of closely related species, and expediting the discovery of previously unidentified organisms in nature^72^. Due to the insufficiency of classical DNA barcodes (*rbc*L, *mat*K, *psb*A*-trn*H, and ITS2) for species identification and phylogenetics in *Stachys*, additional highly variable regions at the genus level as potential markers of* Stachys* should be explored for future identification studies. Based on mVISTA and nucleotide diversity analysis, 5 highly variable regions, including *trn*H-GUG-*psb*A, *trn*K-UUU-*rps*16, *trn*G-UCC-*trn*R-UCU, *trn*N-GUU-*trn*R-ACG, and *rps*12-*trn*V-GAC, were hereby identified, had been validated as reliable markers in previous studies. For example, Fan et al.^20^ found that the amplified fragment of *trn*H-GUG-*psb*A could effectively distinguish plants within the Papaver genus. Yang et al.^73^ used three pairs of primers amplifying variable DNA sequences located in the *psb*A-*trn*K, *psb*B-*psb*H, and *trn*R-*trn*N regions, and their analysis using Maximum Parsimony showed consistent classification and phylogenetic results, making them useful tools for plant species identification and phylogenetic research. Overall, these candidate barcode regions could provide rich molecular marker development information.

### Phylogenetic analysis

Powerful molecular phylogenetics is the basis for establishing stable classifications and provides a solid framework for understanding diverse patterns, historical Biogeography, and trait evolution^74^. The Lamiaceae family, ranked as the sixth largest among angiosperms, serves as a significant reservoir of essential oils, timber, ornamental plants, culinary herbs, and medicinal herbs. This diversity makes it a crucial subject of study in fields such as ecology, ethnobotany, and floristics^75^. In recent studies, *Stachys* has been placed in the tribe Stachydeae within the subfamily Lamioideae, and within Stachydeae, 12 genera and approximately 470 species have been recognized^7^. Stachydeae is the largest and most challenging tribe in the subfamily Lamioideae in terms of classification^16^, which has also been the focus of some previous molecular phylogenetic studies^16,76,77^. These phylogenetic studies are mostly constructed based on gene fragments of Stachydeae or multiple gene fragments of a species to build phylogenetic trees. However, due to the limited number of informative sites, they fail to fully explain the phylogenetic relationships and systematic position of Stachydeae plants^78^. Mounting evidence has indicated the suitability of CP genome sequences for inferring phylogenetic relationships across various taxonomic levels^79^. Using complete CP genomes, many deep level phylogenetic questions have been resolved, such as the determination of the earliest diverging lineages of angiosperms^68,80,81^ or the phylogenetic relationships among *Ferula* species^82^. This approach can better elucidate the complex evolutionary relationships among angiosperms. At the same time, CP genome datasets can also address shallow level phylogenetic questions. In our study, we constructed a phylogenetic tree of 40 Lamioideae plants based on sequence data. The results showed well supported nodes in the phylogenetic tree, and *Stachy* species were not a monophyletic group. This outcome aligned with the overall findings of chloroplast genome-based phylogenetic studies in Lamiaceae^29^. However, previous phylogenetic studies based on chloroplast genomes suggested* S. sylvatica* as a basal branch of the Stachydea system, while *S. byzantina* from West Asia was hereby found to be located at the basal branch of Stachydea. This result was consistent with the study by Xue et al., who utilized ITS + ETS + 5S-NTS for phylogenetic research on *Stachys* species^37^. Furthermore, *S. chamissonis* and *S. coccinea* from North America clustered together with other *Stenogyne*, *Phyllostegia*, and *Haplostachys* plants, while the remaining *Stachys* plants from East Asia formed a clade. This suggested that geographical isolation might have a greater impact on the interspecific relationships within *Stachys*. Overall, the research results offer important implications for the assessment of genetic diversity and systematic phylogenetic studies of *Stachys* in the future. However, this study still failed to fully elucidate the relationships between genera. Additionally, the phylogenetic research was solely based on the chloroplast genome. In this case, the nuclear genome of plants should be further analyzed to comprehensively understand the phylogeny of Stachydeae and even Lamiaceae species, and future studies should also have more genera included. Nevertheless, the phylogenetic research still provides valuable resources for the classification, systematic phylogeny, and evolutionary history of *Stachys*.

## Conclusions

The present research was primarily conducted to assemble the *S. geobombycis* genome and to reannotate the entire CP genome of *Stachys* species. Efforts were made to investigate the characteristics of the cp genome and explore the phylogenetic relationships among *Stachys* plants. The comparative analysis revealed conserved genome size, gene structure, and organization across the *Stachys* species. Furthermore, long repetitive sequences, SSRs, and regions with high variability were identified in the *Stachys* species. Overall, findings serve as a foundation for analyzing genetic diversity, developing mo-lecular markers, and addressing classification and identification challenges within this genus. Additionally, by examining the genetic relationships within *Stachys* species, this research offers comprehensive insights into phylogenetic connections, sheds light on its evolutionary history, and facilitates further research in this field.

## Materials and methods

### Sample collection and DNA extraction

Fresh leaves of *S. geobombycis* were collected from Guangning County, Guangdong Province, China ($21^\circ 38^\prime 49.6^{\prime \prime }$ N, $39^\circ 41^\prime 49.3^{\prime \prime }$ E). This plant is currently preserved at the Guangdong Crop Germplasm Resources Nursery (http://gdseedbank.cn/catalog/guild/). The germplasm resource number is 20224412265. Please contact yongjianluo@foxmail.com for free access. The corresponding GenBank accession number in NCBI is OR327475. Related CP genome sequences were retrieved from the NCBI database. Detailed information about the experimental materials is presented in Table 1. Total DNA was extracted from dried fresh leaves of the samples using a plant DNA extraction kit manufactured by Tiangen Biotech Co., Ltd, and the integrity of the extracted DNA was assessed through 1% agarose gel electrophoresis. Subsequently, the samples were sent to BGI Genomics for further analysis, where the purity and concentration of the total DNA were determined using the NanoDrop 2000 spectrophotometer by Thermo Scientific, USA.

### Library construction and De novo Genome sequencing

MGISEQ-2000 sequencing platform was used to construct a library with an insertion fragment of 500 bp, and paired end sequencing was performed to obtain 150 bp sequences at both ends of each read. Following sequencing, the filtering software SOAPnuke v2.0 33^83^ (https://github.com/ The Beijing Genomics Institute (BGI)-flexlab/SOAPnuke), developed by BGI, was employed for filtering with specific parameters: (1) Adapter trimming: Reads exhibit a 25% or higher match to an adapter sequence were entirely discarded; (2) Low quality filtering: Reads with bases having a quality value below 20 that account for 30% or more of the total read were eliminated; (3) N removal: Reads containing 1% or more N bases with respect to the entire read were removed; (4) Acquisition of clean reads. The resulting data were stored in FASTQ format for subsequent assembly and annotation

### Assembly and annotation of the CP genome

The assembly of the CP genome was conducted using NOVOPlasty v2.7.2 software; the size of k-mers was 39^84^. The gene annotation for the CP genome of *S. geobombycis*, and the downloaded complete CP genomes from NCBI were performed using the default parameters of the Plastid Genome Annotator (PGA) program^40^. Additional manual refinements were carried out using Geneious v11.0.3^41^. Upon the completion of the annotation process, the data were submitted to the NCBI database (https://www.ncbi.nlm.nih.gov/genbank/), and the online tool OGDRAW-DRAW Organelle Genome Maps (https://chlorobox.mpimpgolm.mpg.de/OGDraw.html) was employed visualization of the chloroplast structure.

###  Analysis of contraction and expansion of IR boundaries in CP genomes

The synonymous codon usage in the CP genomes of the three mentioned plants was hereby compared using CodonW v1.42 software (http://codonw.sourceforge.net). The encoded genes were filtered based on the following criteria: (1) The gene’s start codon was ATG. (2) The gene length was $\ge$ 300 bp. (3) Only one gene was selected for genes located in repetitive regions, and pseudogenes were excluded. Meanwhile, the Shuffle-LAGAN mode of the online tool mVISTA (https://genome.lbl.gov/vista/mvista/submit.shtml)^85^ was utilized to perform a comparative analysis of the CP genomes of the 8 plants. MISA software^86^ was used for SSR analysis of the CP genomes, involving parameters including mononucleotide SSRs (repeat unit of 10), dinucleotide SSRs (repeat unit of 6), trinucleotide SSRs (repeat unit of 5), and tetra, penta, and hexanucleotide SSRs (repeat unit of 4). Furthermore, REPuter (https://bibiserv.cebitec.unibielefeld.de/sessionTimeout.jsf)^87^, an online software, was employed for long repetitive sequence analysis of the CP genomes of the 8 plants, and parameters including Hamming Distance of 3 and a minimum repeat unit of 30 base pairs.

### Codon preference and repetitive sequence analysis of the CP genome

In this study, Geneious v11.0.3 software^41^ was used to determine the lengths of the IRa/IRb, LSC, and SSC regions, as well as the boundary genes, in the CP genomes of the *Stachys* species. To visualize and compare the IR boundaries in the CP genomes of 8 *Stachys* species, Adobe Illustrator software was employed for creating comparison maps. For the detection of intra species variations, the mVISTA software was utilized to compare the CP genomes within the *Stachys *species. Meanwhile, Mauve software 110 was applied for the analysis of the homology and collinearity of the CP genome sequences. For calculating nucleotide diversity values, DnaSP v6.0^88^ specifically for the CP genomes of *Stachys* species was used. Finally, the MAFFT v7.487 software^89^ was chosen to perform sequence alignments for all CP genome sequences.

### Phylogenetic analysis

To investigate the placement of *Stachys* within the Lamioideae subfamily and explore relationships between different *Stachys* species, multiple alignments were conducted using complete CP genome sequences from 40 samples representing the Lamioideae subfamily. This comprehensive dataset included representatives from 18 different genera. Besides, the analysis involved the use of several outgroups, including* Lancea hirsuta*, *Lancea tibetica*, *Rehmannia solanifolia*, *Triaenophora shennongjiaensis*, *Wightia speciosissima*, and *Mazus pumilus*. The Phylogenetic trees of complete chloroplast genomes were constructed using the maximum likelihood (ML) method and the Bayesian inference (BI) method. ML analysis was conducted by IQ-TREE (version 2.1.3) in Phylosuite^90,91^ software with a GTR + F + I substitution model and 1000 bootstrap replicates. The Bayesian inference (BI) tree was implemented in MrBayes in Phylosuite^91^ and ran for two million generations in total. Based on the Markov chain Monte Carlo (MCMC) algorithm, the best fitting GTR + F + I substitution model was determined with sampling after every 1000 generations, and the running was stopped once the value of the average standard deviation of split frequencies was less than 0.01 Finally, less than 25% of the aging samples were discarded and a consistent tree was constructed based on the remaining samples.

### Statement of plant collection

 We hereby declare that *Stachys* is not a plant species covered by the IUCN Policy Statement on Research Involving Species at Risk of Extinction and the Convention on the Trade in Endangered Species of Wild Fauna and Flora. The botanical collection work involved in this research has obtained the necessary permits and approvals from relevant local institutions, and strict compliance with applicable laws and guidelines has been ensured. Moreover, we have minimized the impact on the environment and ecosystems during the collection process and made every effort to maintain the survival and reproductive capacity of the *Stachys *plants.

### Supplementary Information

Supplementary Figures.

Supplementary Table S1.

Supplementary Table S2.

## Data Availability

The complete chloroplast genome of *S. geobombycis * generated in this study was submitted to the NCBI database (https://www.ncbi.nlm.nih.gov/) with GeneBank accession number OR327475. The assembled genome sequences and raw sequencing data are accessible in the NCBI database under the research accession PRJNA1076470 with the sample identification number SRR27966668.
