# Supplementary material for: The complete Chloroplast genome of Stachys geobombycis and comparative analysis with related Stachys species
Source: Sci Rep. 2024 Apr 12;14:8523. doi: 10.1038/s41598-024-59132-1 (PMC11014926; doi:10.1038/s41598-024-59132-1)
Supplement: Supplementary file 1 — Supplementary Figures. [file 41598_2024_59132_MOESM1_ESM.docx]

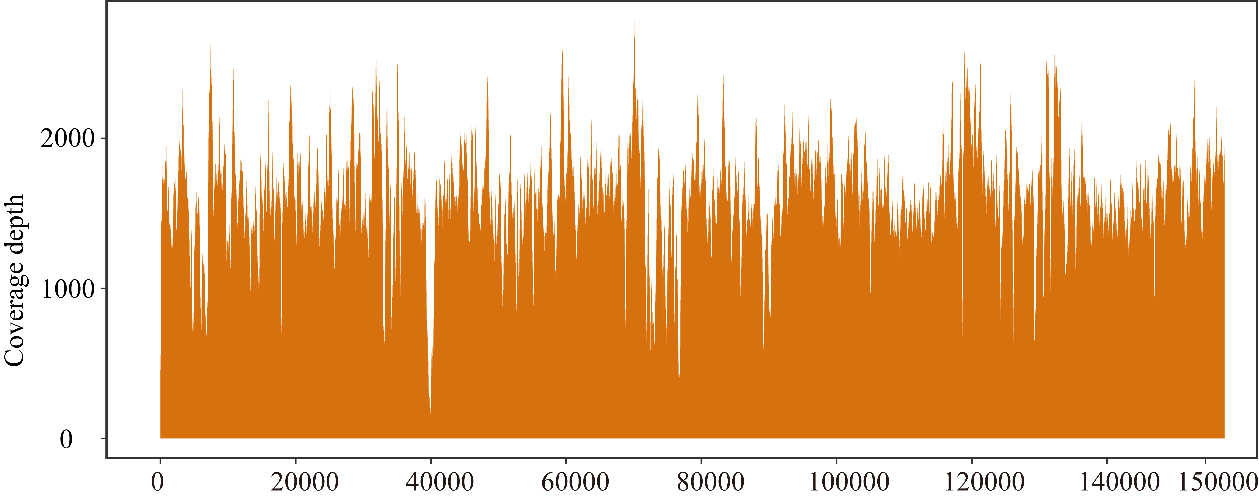


**Figure S1.** Coverage depth figure of the *S. geobombycis* chloroplast genome. The horizontal coordinate is the position of the chloroplast genome, and the vertical coordinate is the sequencing depth.


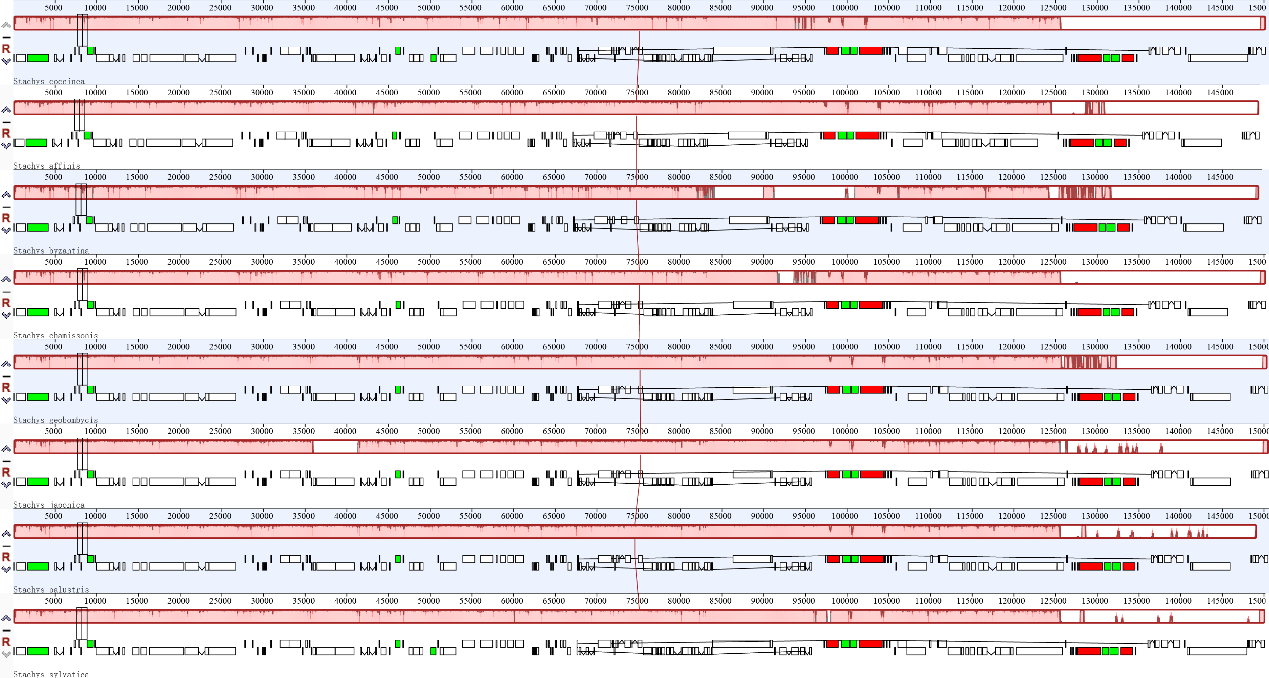


**Figure S2.** Genome rearrangement of 8 taxa of *Stachys*.


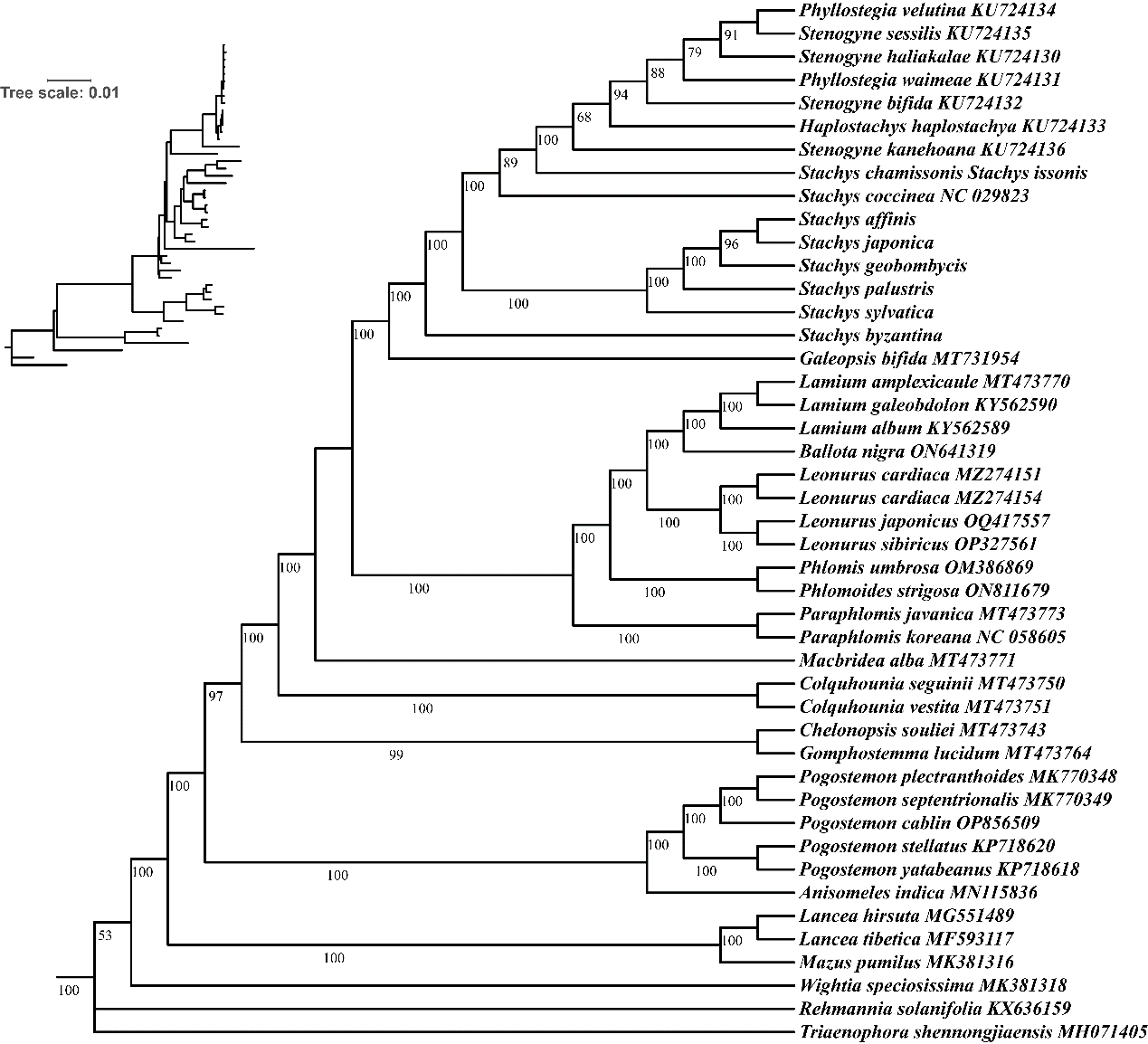


**Figure S3**. Phylogenetic trees inferred from maximum likelihood (ML) analyses based on protein-coding genes. Numbers near the nodes are ML bootstrap support values (BS). The scale bar indicates the number of nucleotide substitutions per site.
